# Transcriptome profiling of two olive cultivars in response to infection by the CoDiRO strain of *Xylella fastidiosa* subsp. *pauca*

**DOI:** 10.1186/s12864-016-2833-9

**Published:** 2016-06-27

**Authors:** Annalisa Giampetruzzi, Massimiliano Morelli, Maria Saponari, Giuliana Loconsole, Michela Chiumenti, Donato Boscia, Vito N. Savino, Giovanni P. Martelli, Pasquale Saldarelli

**Affiliations:** Dipartimento di Scienze del Suolo della Pianta e degli Alimenti, Università degli Studi di Bari Aldo Moro, via Amendola 165/A, Bari, Italy; Consiglio Nazionale delle Ricerche, Istituto per la Protezione Sostenibile delle Piante, SS Bari, via Amendola 122/D, Bari, Italy

**Keywords:** Xylella, *de novo* assembly, Transcriptome, Olive, xylem, Tolerance, Differentially expressed genes, LRR, RLK, ABA

## Abstract

**Background:**

The recent *Xylella fastidiosa* subsp*. pauca* (*Xfp*) outbreak in olive (*Olea europaea*) groves in southern Italy is causing a destructive disease denoted Olive Quick Decline Syndrome (OQDS). Field observations disclosed that *Xfp-*infected plants of cv. Leccino show much milder symptoms, than the more widely grown and highly susceptible cv. Ogliarola salentina. To determine whether these field observations underlie a tolerant condition of cv. Leccino, which could be exploited for lessening the economic impact of the disease on the local olive industry, transcriptional changes occurring in plants of the two cultivars affected by *Xfp* were investigated.

**Results:**

A global quantitative transcriptome profiling comparing susceptible (Ogliarola salentina) and tolerant (Leccino) olive cultivars, infected or not by *Xfp,* was done on messenger RNA (mRNAs) extracted from xylem tissues. The study revealed that 659 and 447 genes were differentially regulated in cvs Leccino and Ogliarola upon *Xfp* infection, respectively, whereas 512 genes were altered when the transcriptome of both infected cultivars was compared. Analysis of these differentially expressed genes (DEGs) shows that the presence of *Xfp* is perceived by the plants of both cultivars, in which it triggers a differential response strongly involving the cell wall. Up-regulation of genes encoding receptor-like kinases (RLK) and receptor-like proteins (RLP) is the predominant response of cv. Leccino, which is missing in cv. Ogliarola salentina. Moreover, both cultivars react with a strong re-modelling of cell wall proteins. These data suggest that *Xfp* elicits a different transcriptome response in the two cultivars, which determines a lower pathogen concentration in cv. Leccino and indicates that this cultivar may harbor genetic constituents and/or regulatory elements which counteract *Xfp* infection.

**Conclusions:**

Collectively these findings suggest that cv. Leccino is endowed with an intrinsic tolerance to *Xfp*, which makes it eligible for further studies aiming at investigating molecular basis and pathways modulating its different defense response.

**Electronic supplementary material:**

The online version of this article (doi:10.1186/s12864-016-2833-9) contains supplementary material, which is available to authorized users.

## Background

*Xylella fastidiosa* (*Xf*) is a polyphagous bacterium causing important diseases of a wide number of crops [1, 2], to which olive (*Olea europaea*) has recently been added. In this last species a strain of *Xf* subsp. *pauca (Xfp)* denoted CoDiRO (abbreviation from the Italian “Complesso del Disseccamento Rapido dell’Olivo”) is associated with a destructive disease named Olive Quick Decline Syndrome (OQDS) [3]. *Xfp* pathogenicity to olive has been recently demonstrated for the CoDiRo strain [4] further underlining the aggressive nature of this bacterium and its potential threat for the Mediterranean and European agriculture.

Being olive a new host, knowledge on the *Xf*-olive pathosystem is lacking, including the aspects concerning the plant response to the pathogen. The final outcome of a *Xf* infection is strongly influenced by the association that the bacterium establishes with the host plant, which could range from commensalism to pathogenicity. Indeed, in several species, *Xf* multiplies and colonizes the host without inducing symptoms, whereas in other, a classical leaf scorching recalling water deficit occurs. Hypotheses behind these symptoms rely on the occlusion of xylem vessels by bacterial colonies aggregated in a biofilm envelope and a plant defense response consisting in the production of tyloses and gums. This species-conditioned behavior, which likely depends on host physiology and/or response to the infection and on the peculiar *Xf* subspecies, is also reflected by the lack of virulence genes and effector proteins, which are present in the genome of other bacterial pathogens, like those encoding the type III secretion system. Because of all the above the current view retains that a disease occurs when the bacterium spreads and multiplies exceeding a certain threshold level and/or finds physiological conditions that make it shifting from a commensalistic relationship to disease induction [5]

A key question regarding *Xf*-plant interactions is to understand whether and how the host perceives a pathogen that colonizes the xylem, a tissue mainly composed, in its ultimate developmental stage, of dead cells. Choi et al*.* [5] demonstrated that xylem-associated cells, particularly those belonging to the protoxylem, sense the presence of the bacterium or of host molecules, i.e. cell-wall degradation products originating from cell wall degrading enzymes (CWDEs) secreted by the pathogen. These authors propose a model in which *Xf*-infected grapevines affected by Pierce's disease specifically perceive the pathogen and respond to its presence by an abscissic acid (ABA)-mediated management of the drought stress imposed by the bacterium. Paradoxically, being ABA a negative regulator of the plant immune response, the possible host defense response is neutralized, thus provoking the establishment of the disease.

In a similar study, aimed at disclosing C*itrus reticulata* resistance to *Xf* [6] by transcriptome profiling, a clear response to the infection was detected, as triggered by the perception of the bacterium, that elicited cell wall remodelling and up-regulation of different hormones (auxin, ABA and jasmonic acid).

In both studies common defense mechanisms were identified consisting on the up-regulation of pattern recognition receptors (PRRs) and the perturbation of cell wall architectural modification and enzymes necessary for biosynthesis of its constituents. Indeed, in grapevine and citrus genes encoding receptor-like kinases (RLK) and cell wall homeostasis are differentially expressed during *Xf* infections.

Plant immune response to pathogens relies on pathogen triggered immunity (PTI) and effector triggered immunity (ETI) [7]. Lack of type III secretion system in *Xf*, which facilitates the entrance of pathogen-secreted effectors into the cell, likely makes PTI the prevailing effective line of defense to this pathogen. PTI effectors are cell-wall-associated PRRs with or without an intracellular kinase domain, which act as signal transducer at the cell wall periphery after perceiving external stimuli. The final outcome of the perception is the activation of a defense cascade to counteract pathogen invasion. Elicitors of PTI are microbe-associated molecular patterns (MAMP) or damage-associated molecular patterns (DAMP), the latter being degradation by-products of the pathogen attack. Known PRRs with a kinase moiety are the FLS2 (flagellin-sensitive 2), the EFR (elongation factor Tu receptor) both encoding RLKs, that recognize the bacterial MAMPs flg22 and Ef-Tu, respectively [8, 9]. A further well-studied RLK is the protein product of the Xa21 gene, which confers resistance of rice to the infection of *Xanthomonas oyzae* pv *oryzae* (*Xoo*) [10]. The most abundant subclass of this large family of proteins counting some 600 and 1000 members in *Arabidopsis* and rice, respectively [11], comprises leucine-rich repeats (LRR)-RLKs, characterized by the presence of diverse extracellular protruding motifs rich in leucine and other hydrophobic amino acids. This diversity is thought to originate from evolutionary events in response to the necessity of binding to various MAMP or DAMP ligands [12].

Important events aiming at counteracting microbe attack occur at the cell wall periphery. This first pre-existing barrier undergoes an intense remodelling during biotic or abiotic stresses to maintain homeostasis and transfer external stimuli to the nucleus. Indeed, during water stress, which is the main effect of *Xf* infections, the involvement of cell wall is necessary to adapt cell turgor and expansion and preserve xylem conductivity. A further role of cell wall is related to its polysaccharide composition and its susceptibility/resistance to microbial aggression. Major *Xf* virulence factors are CWDEs polygalacturonase (PG) and endo-1,4-β-glucanase (EGase) [13], which likely digest homogalacturonan (HG) and xyloglucan (XyG), two major components of plant cell-walls [14, 15]. The methyl esterification status of HGs, which is tightly regulated by pectin methylesterase (PME) enzymes and corresponding PME inhibitor (PMEIs) proteins, influences its susceptibility to microbial PGs. Notably, highly esterified HGs were found in intervessel pit membranes of grapevine genotypes showing resistance to *Xf* [16] whereas a down regulation of CWDEs PG and pectate lyases is observed in the resistant Ponkan mandarin (*Citrus reticulata*) during *Xf* infection [6]. Maintaining cell wall integrity is part of a defense mechanism that, by regulating the response to external stimuli and its impairment during pathogen attack, results in the release of DAMPs, i.e. the molecular constituents of membranes acting as regulators of plant immune response [17–20].

A possible strategy for the management of *Xf-*induced diseases is the identification of cultivars showing tolerance/resistance to the pathogen and their use in the field or in breeding programs. Field observations in OQDS-affected areas have repeatedly shown that plants of the olive cv. Leccino vegetate nicely and exhibit very mild symptoms, contrary to those of cv. Ogliarola salentina which are heavily damaged and succumb to infection. Specifically, the canopy of the OQDS-affected trees of the cv. Ogliarola salentina shows a progressive and complete desiccation, whereas in the cv. Leccino these phenomena are limited to few scattered twigs or distal branches. Indeed, a preliminary rough investigation, showed that there is much less bacterium in the xylem and leaf tissues of cv. Leccino than in those of cv. Ogliarola salentina [21, 22]. To investigate the molecular mechanisms underlying these differential phenotypes and to gain knowledge on their response to infection, a transcriptome profiling of *Xfp*-infected plants of both cultivars was undertaken.

## Results

### The population size of *Xylella fastidiosa* in cv. Leccino plants is lower than in Ogliarola salentina

**Fig. 1:**
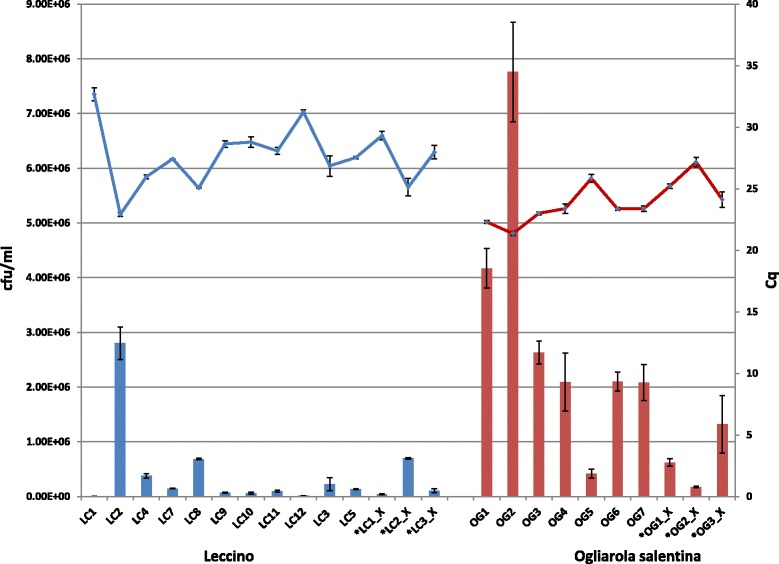
Population sizes of *Xylella fastidiosa* in fourteen and ten different plants of cv Leccino (LC) and Ogliarola salentina (OG). Population sizes (bars: CFU/ml) were inferred by *Cqs* (lines) obtained in qPCR using a previously constructed calibration curve. LC1_X, LC2_X, LC3_X, OG1_X, OG2_X and OG3_X correspond to xylem tissues used to synthesize the mRNA libraries

As mentioned, *Xfp* qPCR detection assays had shown that cv. Leccino has a lower pathogen population than cv. Ogliarola salentina. To confirm these findings a calibration curve was constructed correlating known amounts of *Xfp* cells (CFU/ml) to their corresponding *Cqs* obtained by qPCR assays (*not shown*). Bacterial titer determined from the calibration curve showed that 14 cv. Leccino plants had CFU/ml in the range 4.81E + 3 - 2.80E + 6 with an average of 3.89E + 4 whereas in 10 cv. Ogliarola salentina plants the titer was 1.74E + 05 and 7.76E + 06 with an average of 2.33E + 6 (Fig. 1). This confirmed that the size of *Xfp* population is two orders of magnitude lower (E + 04 vs E + 06) in cv. Leccino than in cv. Ogliarola salentina.

### Transcriptomes of cvs Leccino and Ogliarola salentina plants

Ten mRNA libraries were sequenced from three biological replicates of Leccino (LC_X) and Ogliarola salentina (OG_X) infected plants (Table 1) and two biological replicates of the corresponding healthy cvs (LC_H and OG_H). More than 240 million raw reads were produced from the 10 libraries, which each had 18 to 36 million sequences, that, after filtering for rRNA and adapters, yielded 13 to 35 million of high quality sequences.

**Table 1:** Summary of Illumina sequencing data

Leccino					
Condition	*Xfp* infected			Healthy	
Sample ID	LC1_X	LC2_X	LC3_X	LC5_H	LC6_H
Raw reads	18,555,271	21,787,854	20,796,032	21,103,537	22,944,263
rRNA/adapter filtered reads	13,179,688	21,566,586	20,693,350	20,895,346	22,130,293
Ogliarola salentina					
Condition	*Xfp* infected			Healthy	
Sample ID	OG1_X	OG2_X	OG3_X	OG5_H	OG6_H
Raw reads	35,105,651	36,636,150	22,406,467	22,540,501	20,364,603
rRNA/adapter filtered reads	34,841,076	36,204,457	22,054,765	22,471,506	20,301,798

Because the olive genome is not yet available to the public, three databases containing transcripts sequences were reconstructed from the RNASeq data using a *de novo* assembling strategy. Databases obtained from the five (three infected and two healthy plants) cvs Leccino and Ogliarola salentina libraries, had a total of 279,855 and 354,422 unique transcripts larger than 100 bp, respectively. Successively, a database was generated by merging non-redundant transcripts from the two cultivars to obtain an “olive xylem transcriptome”. This combined database contained 370,495 unique transcripts with a maximum length of 8,979 bp (Table 2; Additional file 1). These databases of transcripts were used as references to align reads and quantify gene expression.

**Table 2:** *De-novo* assembly statistics. Summary of transcriptome data *de novo* assembled from libraries of cvs Leccino and Ogliarola salentina. The combined transcriptome was obtained by merging non-redundant transcripts of the two cultivars

Cultivar	Leccino	Ogliarola salentina	Combined
Total reads	98,465,263	135,873,602	–
Number of RNAseq libraries	5	5	10
Minimum length of transcript (bp)	100	100	100
Maximum length of transcript (bp)	8,979	7,860	8,979
Average length of transcripts (bp)	308	313	270
Total length of sequence (bp)	86,372,337	111,135,716	100,071,628
Total number of sequences	279,855	354,422	370,495
N50 stats	> = 781 bp	> = 850 bp	> = 757 bp
N75 stats	> = 406 bp	> = 424 bp	> = 328 bp
GC percentage %	41.73 %	40.75 %	40.61 %

The genome representativeness of the “olive xylem transcriptome” was validated by the CEGMA pipeline [23], which searches for the presence of a set of core eukaryotic genes (CEGs). The CEGMA analysis showed that 236 out of 248 CEGs (95.1 %) were detected in our set of transcripts and that 196 (79 %) of them were almost 70 % of the length of the matched protein (Additional file 2). The four groups of CEGs were highly represented in our transcripts database since a minimum of 94.64 % transcripts matched CEG group 2. This assessment increased the level of confidence that the quality of the olive transcriptome assembly was informative enough to proceed with a gene expression analysis. Moreover, the suitability of the obtained assemblies in describing differential gene expression was shown by the high percentages of reads from each library that align to the assembly, which ranged from 89 to 93 % (*not shown*).

### Differential expression analyses

**Fig. 2:**
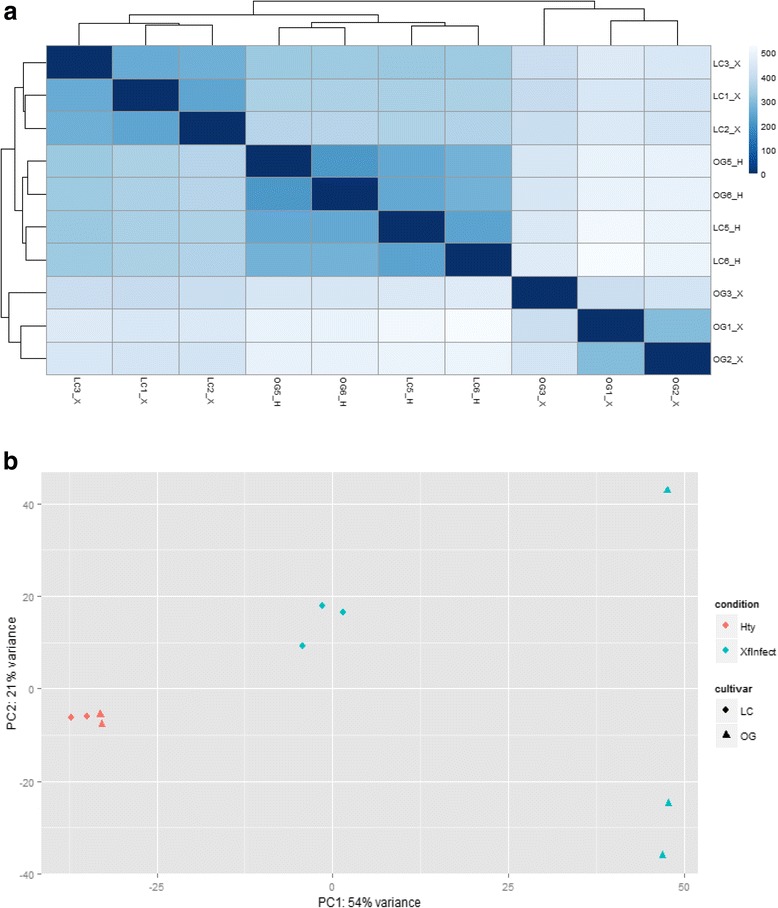
Statistical analysis of libraries using rlog transformed data: **a**) Heatmap showing hierarchical clustering of biological replicates using sample-to-sample distances. Blue and white colors represent low and high values of distance, respectively. **b**) Principal component analysis (PCA) showing sample clustering of libraries. Different shape of data points refer to the cv Leccino (LC) and Ogliarola salentina (OG) whereas red and blue colours indicate healthy (Hty) and Xylella fastidiosa-infected (Xfinfect) plants, respectively

A distance tree obtained by a statistical comparison of r-log transformed raw reads from each library, identified four well-defined clusters containing the three infected cv. Leccino, the three infected cv. Ogliarola salentina, the two healthy cv. Leccino and the two healthy cv. Ogliarola salentina plants, respectively (Fig. 2a). Significantly, the topology of the distance tree showed clustering of all healthy and infected cv. Leccino plants and locates more distantly the infected cv. Ogliarola salentina olives. Each group is clearly separated from the other. Further statistical evaluation of the relationships occurring among analyzed plants and correlations among biological replicates are shown by principal component analysis (PCA) (Fig. 2b), which clearly groups healthy or infected replicates and further confirm the distant relationship of infected cv. Ogliarola salentina plants with the other tested plants. Both methods showed that groups of replicated libraries are highly correlated with a limited deviation of a single infected cv. Ogliarola salentina plant in PCA analysis, which, however, are all undoubtedly separated from the healthy plants of both cvs and from infected cv. Leccino olives. Relevant results of this initial analysis are: (i) a distinct clustering according to the healthy/infected status; (ii) an extremely diverse transcriptome profiling of the two infected cultivars; (iii) a similar transcriptome profile of healthy plants, regardless of the cultivar to which they belong. This latter finding denotes a similar gene expression pattern in the two cultivars. In summary, the outcome of these preliminary studies suggests that the two cultivars react differently to *Xfp* infection.

#### Analysis of differential gene expression in the xylem of cv. Leccino infected by Xfp

**Fig. 3:**
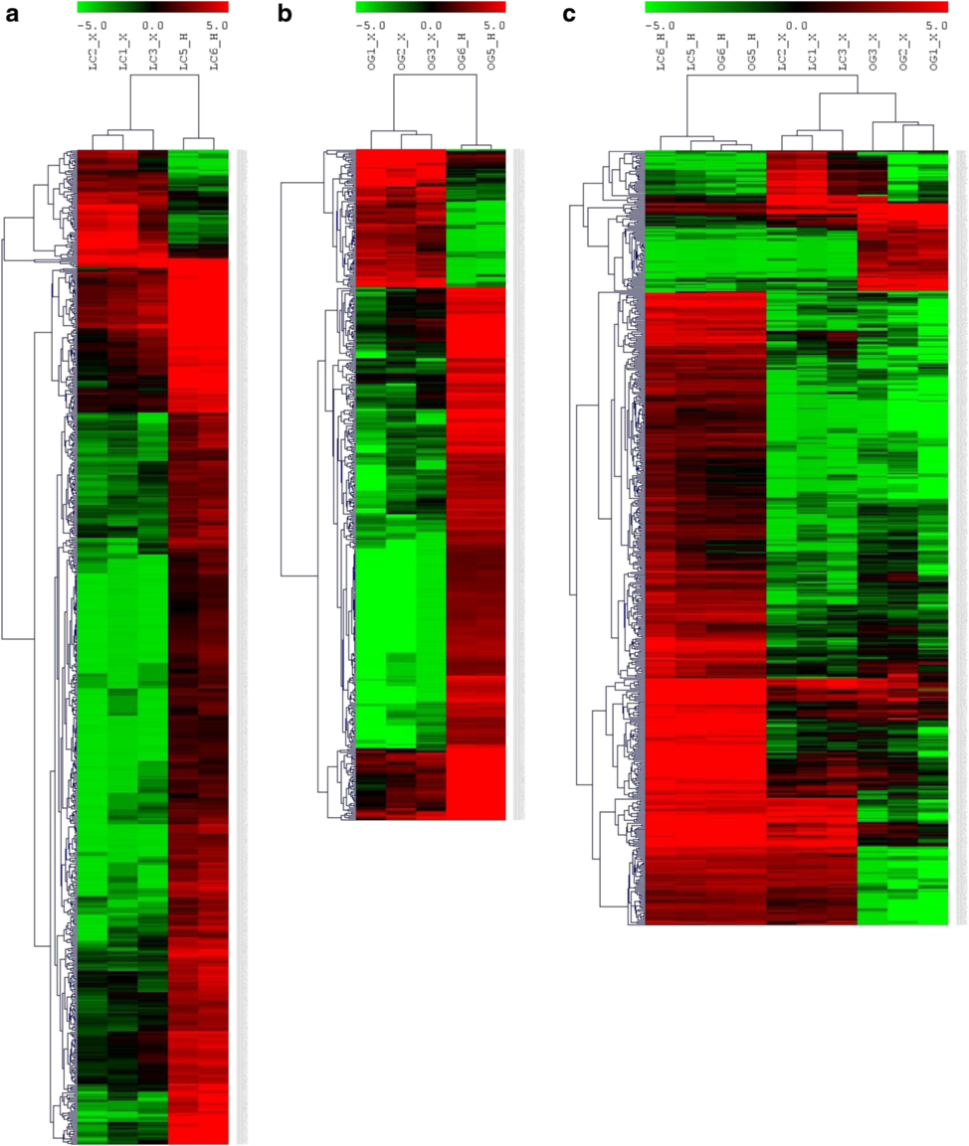
Heat map showing the 659 (**a**), 447 (**b**) and 512 (**c**) differential expressed transcripts of the Xf-infected cv. Leccino (LC1_X; LC2_X; LC3_X) vs healthy (LC5_H; LC6_H), the Xf-infected cv. Ogliarola salentina (OG1_X; OG2_X; OG3_X) vs healthy (OG5_H; OG6_H) and all cv Leccino and Ogliarola salentina plants, respectively. Fold expression values are indicated by different colours. Transcripts alignment was performed on the corresponding cv Leccino (**a**), Ogliarola salentina (**b**) and combined (**c**) transcripts databases

A strong perturbation of the transcriptome of cv. Leccino was induced during *Xfp* infection as visualized by the corresponding heat map showing fold values of differentially expressed transcripts (Fig. 3a; Additional file 3). The cultivar perceived the pathogen presence showing a high number (659) of transcripts undergoing a differential expression in infected plants. The majority (586) of differentially expressed genes (DEGs) was down-regulated whereas only 73 were over expressed.

**Fig. 4:**
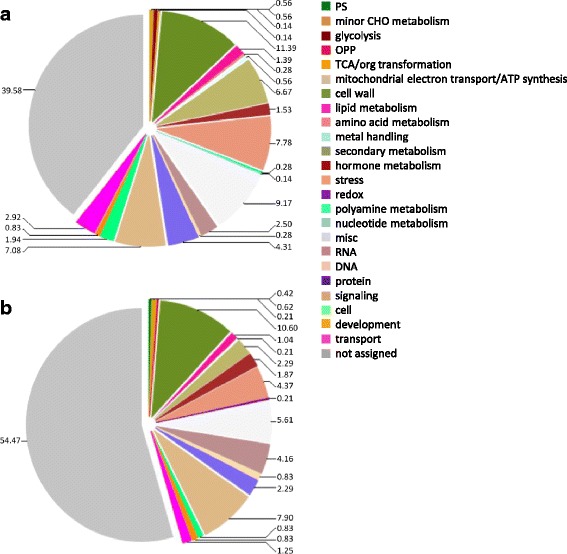
Categorization of DEGs varying in the cvs Leccino (**a**) and Ogliarola salentina (**b**) infected by *Xfp*. Assignment to protein functional classes, expressed as percentages out of the total proteins, was obtained by Mercator and MapMan v.3.5.1

Categorization of these DEGs into functional plant categories by Mercator (Fig. 4a; Additional file 3) showed homologies to plant proteins or protein signatures of cell wall (11.39 %), stress (7.78 %) and signaling (7.08 %) classes, which were among the most destabilized in cv. Leccino upon *Xfp* infection, in comparison to healthy plants of the same cultivar. A large class (9.17 %) of miscellaneous transcripts is differentially expressed and, as expected because of the lack of an annotated olive genome, 39.58 % of the transcripts were not assigned to any plant protein class.

In depth analysis revealed a substantial involvement of cell wall proteins in the response to *Xfp*, which is demonstrated by the down-regulation of 82 DEGs (Additional file 3). The majority of these DEGs, whose orthologues regulate cell wall integrity in *Arabidopsis thaliana* and other species, undergo a significant decrease (from two to eight fold) of expression in infected plants. The protein functions involved, concern cell wall biosynthesis, architectural organization, cell wall degradation and adhesion. Many of these DEGs, encoding integral membrane proteins with an extracellular domain, are similarly repressed in *Xf*-resistant Ponkan mandarin [6]. Several down-regulated DEGs encode orthologues of the *A. thaliana* Fasciclin-like arabinogalactan-proteins (FLA) 11 and 12 (at5g03170; at5g60490). FLA 11 and 12 regulate cellulose deposition having a direct effect in cell wall integrity, strength and adhesion [24]. Many down-regulated transcripts which are orthologous to *A. thaliana* pectinesterases (PMEs; at5g53370), pectinesterase inhibitors (PMEIs; at3g49220; at5g09760), pectin lyases (PL; at5g19730; at1g60590; at1g48100; at1g10640; at5g48900; at3g61490) and polygalacturonase (PG; at1g70370) control pectin esterification level and degradation, which are important processes in cell wall development and defense response.

Transcripts orthologous to *A. thaliana* genes related to secondary metabolism encoding laccases (at2g40370; at2g3021; at2g38080; at5g01190; at5g60020) are also strongly (up to sevenfold) down-regulated.

Overall, the analysis of down-regulated transcripts of cv. Leccino indicates that the cell wall undergoes a strong re-modelling and the secondary metabolism is affected by *Xfp* infection.

Several genes responding to biotic stresses or having a signaling function (Additional file 3) are present among up-regulated transcripts. DEGs showing homology with receptor-like proteins, involved in signal transduction and defense response in different plant species, are the protein functional class most represented. These transcripts, encoding leucine-rich repeat (LRR) proteins, either possessing a kinase domain. (i.e. LRR-RLK receptor-like kinase; at5g49290; at1g74190; at1g74180; at3g23120; at5g27060) or not (i.e. LRR-RLP Receptor-like proteins; at1g07390; at1g74170; at3g53240), are induced up to fourfold in *Xfp*-infected cv. Leccino plants.

The portrayal arising from this analysis suggests that the cv. Leccino perceives *Xf* infection at the cell wall level and likely commits this cell district to counteract the pathogen attack. This commitment translates into modulating the cell wall architecture and polysaccharide composition, and in up-regulating several members of the LRR-RLK and LRR-RLP classes of proteins.

#### Analysis of differential gene expression in the xylem of cv. Ogliarola salentina infected by Xfp

*Xfp* infection induces the differential expression of 447 genes in susceptible cv. Ogliarola salentina, 92 of which are up- and 355 down-regulated (Fig. 3b).

The main categories of DEGs consist of cell wall (10.60 %), signaling (7.9 %) and stress (4.37 %) classes of proteins. For a large fraction of transcripts (54.47 %) no match with functional classes of plant proteins was found in databases using the Mercator software (Fig. 4b).

Genes encoding cell wall proteins involved in architectural organization and degradation (PGs; PLs; PMEs; PMEIs) are down-regulated, as observed in cv. Leccino (Additional file 3).

Cell wall orthologous proteins of *A. thaliana* (at4g17030 and at4g30380) belonging to the expansin family are exclusively up-regulated in cv. Ogliarola salentina. These proteins, classified in four families, expansin A (EXPA), expansin B (EXPB), expansin-like A (EXLA) and expansin-like B (EXLB) [25], regulate cell expansion through cell-wall loosening.

Further up-regulated transcripts encode proteins orthologous to G-type lectin S-receptor-like serine/threonine-protein kinase SD3-1 (og_xylem_225531) of African oil palm (*Elaeis guineensis*), *A. thaliana* short-chain dehydrogenase/reductase (OG_xylem_197839) (at1g52340), early light-induced proteins (ELIP) (at4g14690) and far-red impaired response 1 (FAR-RED) (at4g15090) in different plant species.

Significantly, LRR-RLKs and LRR-RLPs genes are down-regulated in cv. Ogliarola salentina (Additional file 4) and, according to BLASTX analysis, several of them are probably inactive. By contrast, a couple of transcripts encoding orthologous proteins of cysteine-rich RLKs (CRK) are strongly (CRK8/10, at4g23180.1) up- or down- (CRK6, at4g23140.1) regulated. Both proteins have been associated with a response to biotic stresses in wheat [26] and *A. thaliana* [27].

#### Analysis of differential gene expression between healthy cvs Leccino and Ogliarola salentina

Comparison of transcriptomes of healthy cultivars could allow for the identification of possible pre-existing mechanism of resistance to *Xf*. Eighty-six genes were differentially expressed in this assessment, which is the lowest number of transcripts observed throughout this study (Additional file 5). This finding indicates that the two cultivars do not differ much in the expressed genes, thus confirming the clustering of healthy plants observed in the statistical analysis (Fig. 2). A clear predominance of protein class function was not observed among altered transcripts, as it could be expected in the absence of biotic or abiotic stresses. The range of transcript fold expression, spanning from 2.9 to 3.9, is moderate, as compared with those observed in *Xfp*-infected plants. This is then taken as an evidence that: (i) mechanisms of resistance to *Xfp* do not pre-exist in healthy plants; (ii) the basal transcriptomes of the two cultivars are similar and (iii) further confirms that the observed differential expression in diseased plants is a specific response to *Xfp* infection.

#### Analysis of differential gene expression between Xfp-infected cvs Leccino and Ogliarola salentina

**Fig. 5:**
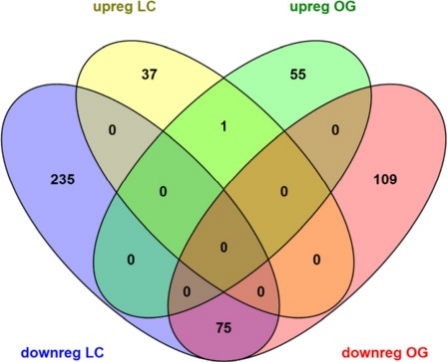
Venn diagram showing differentially expressed genes between *Xfp*-infected cvs Leccino (LC) and Ogliarola salentina (OG). Up- (upreg) and down-regulated (downreg) genes are shown in the Venn diagram using different colours

Differential gene expression between *Xfp*-infected plants of cvs Leccino and Ogliarola salentina was studied using our “olive xylem transcriptome” database. Comparison between transcriptomes of infected plants of the two cultivars showed clearcut incompatible profiles (Fig. 3c; Fig. 5; Table 3). Particularly, 38 and 56 genes were exclusively up-regulated in cvs Leccino and Ogliarola salentina, respectively and only one was common between the two groups (Fig. 5; Additional file 6). By converse, 235 and 109 genes are down-regulated in cvs Leccino and Ogliarola salentina respectively, while 75 are common.

**Table 3:** Differential Expressed Gene (DEGs). Numbers of DEGs obtained by DESeq2 and successively filtered by SeqMonk. Common: common DEGs between DESeq2 and SeqMonk analysis

	deg LC	deg OG	deg OG LC Hty
STATISTIC	LC XInf vs LC Hty	OG XInf vs OG Hty	LC Hty vs OG Hty
DESeq2	4165	5851	951
SeqMonk	407	293	186
common	348	240	86
upreg	38	56	–
downreg	310	184	–

The majority of the 38 up-regulated transcripts of cv. Leccino are orthologues of LRR-RLK proteins of different plant species (Additional file 6). Among the up-regulated transcripts of cv. Leccino there are orthologues of the predicted chaperone DNAJ6 of *Sesamum indicum* (OG_xylem_325091) and of a *A. thaliana* NAC transcription factor (at1g69490). The single up-regulated transcript, common to both cvs, was an orthologue of the stachyose synthase-like gene of *Sesamum indicum* (ipr008811), whose signaling role in response to biotic stress is debated [28].

The 310 cv. Leccino and 184 cv. Ogliarola salentina down-regulated transcripts (Additional file 6) reproduce the differential gene expression previously observed in intra-cultivars comparisons. The majority of repressed genes, considering also the 75 which are common to both cultivars, encodes proteins involved in cell wall remodelling and integrity, highlighting the major role of this cell compartment in the response to the *Xf* stimulus either for signaling or protective functions.

Orthologues of Expansin-like B1 (OG_xylem_175154; OG_xylem_146321) and ELIPs (LCxylem_81506; OG_xylem_197731) genes are present among the 56 up-regulated transcripts in cv. Ogliarola salentina. In this latter list an orthologue of a dehydrin of *Rhododendron catawbiense* (OG_xylem_111461) and a late embryogenesis abundant protein (LEA) of *A. thaliana* (OG_xylem_45532) were found. Dehydrins belong to the family of LEA, i.e. proteins induced during extreme stresses caused by drying, cold and salt excess. Their main role is to stabilize enzymes or membranes during these stresses [29]. It should be noted that three proteins showed homologies to members of the LEA family among the cv. Ogliarola salentina down-regulated transcripts (LCxylem_268715, LCxylem_237449 and LCxylem_206556) (Additional file 6). However, according to Hincha and Thalhammer [29], these proteins are erroneously classified as LEA since they have a high glycine content thus are likely to be hydrophilines.

### GO enrichment analysis

A reference set of 22,373 transcripts was selected for GO enrichment analysis to identify genes and pathways involved in the response of cvs Leccino and Ogliarola salentina to *Xfp* infections. Of these 20,668 found a hit by BLASTX search. Blast2GO software allowed to functionally annotate and assign a GO term to only 5,901 out of 22,373 transcripts. DEGs up- and down-regulated in cvs Leccino and Ogliarola salentina (Fig. 5) were tested for GO enrichment analysis, although the low number of the annotated transcripts available limited the study.

GO terms at the cellular component (C) ontology level, “membrane”, integral component of membrane”, “intrinsic component of membrane” and “membrane part” (Additional file 7; sheet 38 DEG) were over represented among those up-regulated in cv. Leccino, which confirms the involvement of the cell wall components in the signaling response to *Xfp*. Conversely, in this cv. the molecular function (F) ontology level “catalytic activity” (Additional file 7; sheet 235 DEG) has many over-represented terms referred to the cell wall re-modeling and structural organization. Similar GO terms (i.e. “cell wall modification”, “cell wall organization”, “cell wall organization and biogenesis”) at the F and P (biological process) ontology levels (Additional file 7; sheet 109 DEG) are over-represented among the 109 down-regulated DEGs in the cv. Ogliarola salentina.

### RT-qPCR validation of selected genes and correlation with RNASeq

Validation of differentially expressed genes was performed by RT-qPCR. Selection of genes whose expression did not vary among healthy or *Xfp*-infected plants, to be used as references in RT-qPCR, was made based on transcriptome data. The expression was evaluated of genes currently used in RT-qPCR analyses (Additional file 8a; Additional file 9) such as polyubiquitin (UBQ), elongation factor 1-alpha (EF1α), elongation factor 2-like (EF2), ubiquitin-conjugating enzyme E2 (UBCE) and beta actin (β-ACT). Their transcripts showed homologies with *Olea europea* Unigenes in the Transcript Shotgun Assembly (TSA) database. RT-qPCR assays showed that polyubiquitin (UBQ) was the most stable transcript in healthy and infected cultivars. In addition, since UBQ showed the highest efficiency and the lowest *Cq* values in all replicates (*not shown*), it was selected as a suitable reference gene to normalize analyses of gene expression.

**Fig. 6:**
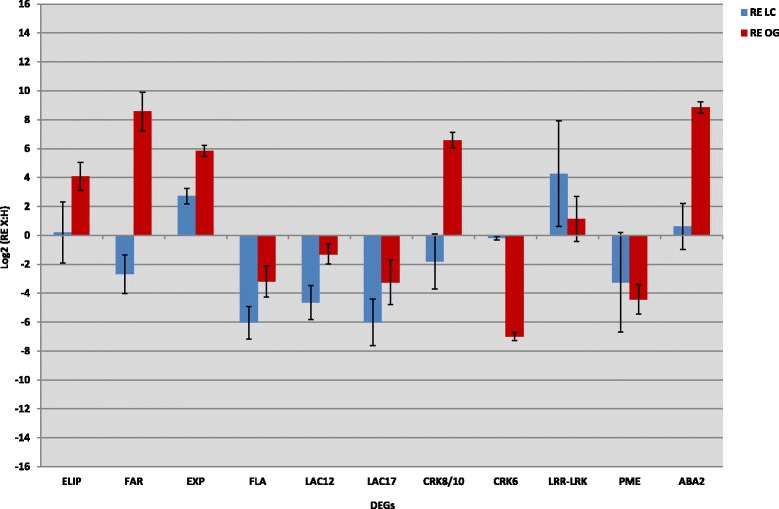
RT-qPCR expression analysis of differentially expressed genes. Log2 fold expression values of eleven RNASeq-selected genes in the cvs Leccino (LC) and Ogliarola salentina (OG). RT-qPCR reactions were done in triplicate and fold values of infected (X) *vs* healthy (H) plants were calculated using the ∆∆Ct method

The expression of 11 genes, selected according to the relevant pathways involved in olive response to *Xfp*, was studied (Additional file 8b). Selection of these genes was made based on expression data from transcriptome analysis. Besides the already discussed DEGs, (ELIP, FAR-RED, Exp, FLA, LAC17, LAC12, ABA2, LRR-LRK, CRK8/10 and CRK6) an orthologue of a pectinesterase 3-like (PME: Additional file 9) was selected since it was down-regulated only in *Xfp*-infected cv. Ogliarola salentina plants (Additional file 8b). RT-qPCR transcriptional changes of all these genes (Fig. 6), perfectly fitted the RNASeq data. In particular, transcripts encoding proteins responding to drought (ELIP, FAR and ABA2) were all over expressed in cv. Ogliarola salentina, whereas up-regulation of a selected LRR-RLK was confirmed in cv. Leccino. Conversely, the observed differential expression of CRK8/10 and CRK6 in both cvs was confirmed by RT-qPCR. Alteration of transcripts related to cell wall remodelling in response to stimuli is represented by transcripts encoding two laccases (LAC12 and LAC17), both down-regulated in cv. Leccino, and expansin (EXP), which is up-regulated in cv. Ogliarola salentina. When a one-way ANOVA was performed to statistically evaluate RT-qPCR data of single transcripts (Additional file 10), significant differences of the expression levels of the ELIP, FAR, EXP, FLA, LAC17, PME, ABA2 and CRK6 were found among the four groups. Post hoc multiple comparisons using the Tukey HSD test showed that *Xfp* infection induced statistically significant differences in transcripts expression levels either intra-cultivar (LC_X *vs* LC_H and OG_X *vs* OG_H) or between cultivars (LC_X *vs* OG_X).

**Fig. 7:**
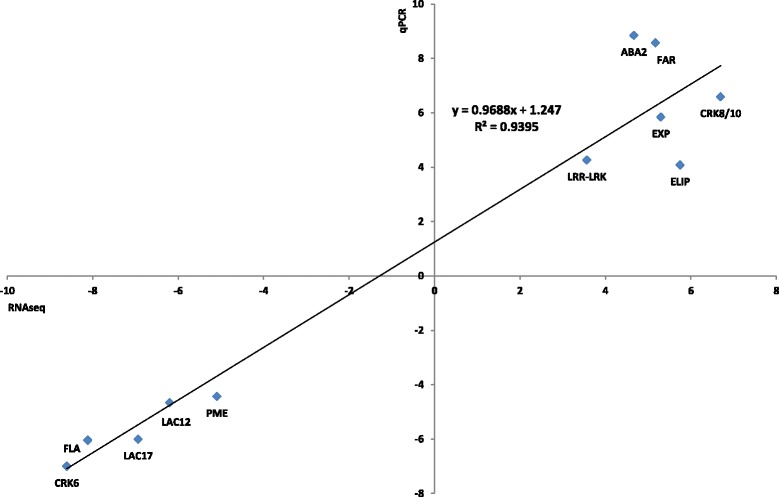
Correlation of RNA-seq and real time RT-PCR. Correlation plots indicate the relationship between RT-qPCR results [fold change as Log2 (Exp X/H); Y-axis] of eleven selected transcripts and the corresponding data from RNA-seq analysis (Log2 fold change from DESeq2; X-axis)

A further confirmation of the agreement between transcriptome and RT-qPCR data came from linear regression correlating Log2 fold expression values obtained by the two techniques (Fig. 7). A line calculated by the interpolation of these data has a high coefficient of determination (R^2^ of 0.9395) demonstrating the reliability of the differential expression analysis.

## Discussion

Olive infection by *Xfp* strain CoDiRO and its association with OQDS constitutes a new pathosystem. Infections on olives have been first reported in California [30]. However, the *Xf* subspecies isolated from olive in California and Italy belong to the taxa *multiplex* and *pauca*, respectively, thus making the comparison inappropriate, since different subspecies have diverse host range and/or pathogenicity [31, 32]. By converse *Xfp* outbreaks in olive, characterized by the same symptomatology observed in Apulia have been recently reported from Argentina [33] and Brazil [34].

After several attempts to reproduce symptoms with artificial inoculation of *Xfp* in olive plants, only recently it was shown that the bacterium is able to systemically infect olive plants of different cultivars, and to induce consistent symptoms of desiccation in the most susceptible cultivar as soon as 13-14 months post inoculation (http://www.efsa.europa.eu/en/press/news/160329) [4]. This long time requested for symptom appearance could likely depend on the time needed to reach, according to a current infection model [16, 35], a bacterial threshold beyond which the disease develops. In addition, the polygalacturonase of the *Xfp* strain CoDiRO has a premature stop codon [36], which could be responsible, as already proposed for the 9a5c CVC subspecies *pauca*, for the slow movement of the bacterium and its low titer in the host [37]. Moreover, additional reasons that induced to study *Xfp*/olive interactions in adult plants have been field observations of the milder OQDS symptoms shown by cv. Leccino with respect to the cv. Ogliarola salentina.

This study shows that *Xfp* reaches a lower titer in the stem of the tolerant cv. Leccino than in cv. Ogliarola salentina. A positive correlation between bacterial population size and expression of symptoms of Pierce’s disease was reported for susceptible and resistant grapevine cultivars [35, 38, 39]. In these studies [35, 37] the mechanism of grapevine resistance is supposed to operate in the xylem vessels of the stem by inhibiting bacterial multiplication and movement. Likewise, in resistant citrus genotypes *Xf* is restricted to the primary xylem of the stem where an active plant response occurs, which culminates in the lignification and successive impairment of bacterial colonization [37]. Since in olive the bacterial titer was evaluated in xylem tissues of the stem, we suggest that the multiplication of *Xfp* CoDiRO is impaired in this cv. Leccino setting.

This study shows clearly distinguishable transcriptional changes in *Xfp*-infected *vs* healthy olives as also supported by statistical analysis. Healthy plants of the two cultivars have similar transcriptome profiles whereas those of the corresponding *Xfp*-infected olives group in diverse clusters. Interestingly, PCA analysis groups the transcriptomes of *Xfp*-infected plants of the tolerant cv. Leccino more closer to those of the healthy plants of both cultivars than to those of the susceptible cv. Ogliarola salentina.

Two major outcomes arise from transcriptome profiling of *Xfp*-infected olives: (i) the consistent involvement of the plant cell wall; (ii) the specific response of cv. Ogliarola salentina to the water stress induced by the pathogen.

The comparable transcriptome profiles of healthy plants, characterized by the smallest number (86) of DEGs among all the analyzed comparisons, and the significant transcriptional changes in the infected cultivars, indicate that olives perceive the infection of *Xfp*. As mentioned by Choi et al. [5], living cells of the protoxylem could be responsible for this perception. Although originating from asynchronous infections, the tissues analyzed in the present work were collected from healthy looking shoots close to branches with symptoms of leaf scorching and dieback. In addition, since *Xfp* was detected in the same tissues used to generate the mRNA libraries, it is presumable that these would be able to sense the pathogen and/or its components (MAMPs) or the damages caused by the infection (DAMPs). The observation that transcripts categorized in the “cell wall” class of protein functions did not occur in the comparison between the healthy controls of the two cultivars, strongly supports the assumption that this cell district perceives, directly or indirectly, the presence of the bacterium.

Both *Xfp*-infected cultivars down-regulate genes involved in controlling the esterification level (PMEs, PMEIs) and degradation processes (PGs, PLs) of cell wall pectins, as it was observed in Ponkan mandarin resistant to *Xf* infection [6]. Besides its known role in plant immunity, an intriguing outcome of the cell wall degradation processes, could be the ability of plant pectins to up-regulate the rpfF gene, which encodes the *Xf* diffusible signaling factor (DSF), as reported by Killiny and Almeida [40]. In this framework, the modification of the polysaccharide content in the xylem could modulate *Xf* gene expression, although indirectly.

The observed down-regulation of two laccase genes in *Xfp*-infected cv Leccino contrasts with the report of their induction in the *Xf*-resistant Ponkan mandarin [6, 37] since lignification is supposed to hinder bacterial movement. However, bacterial colonization in the resistant mandarin is restricted to the primary xylem in which *Xf* is trapped. Conversely *Xfp* moves systemically in cv. Leccino in which these preliminary data indicate that the mechanism of resistance likely consists in a reduced multiplication rate. Although the role of these enzymes is not clear, a study in poplar reports their implication in maintaining the cell wall structure and integrity of xylem fibers [41]. Moreover, the down-regulation of a laccase transcript (LAC17) was observed in *A. thaliana* upon *Botrytis cinerea* and *Turnip mosaic virus* (TuMV) infections [42].

The activation of a cell wall surveillance system was shown by the down-regulation of DEGs orthologous to *A. thaliana* COBRA-like proteins (at5g15630) observed in cv. Leccino. Repression of these proteins may alter cellulose deposition and oriented cell expansion. Particularly, loss-of-function mutants of this gene strongly induce a defense response in *A. thaliana*, functioning as a cell wall-located surveillance system [43]. The outcome of this mutation is the accumulation of stress-induced anthocyanins and the deposition of callose resulting in an anti-bacterial action. Similarly, FLA 11 and 12 have a role as signaling molecules in response to various biotic and abiotic stresses. A recent paper reports the high concentration of these proteins in xylem sap of cotton [44]. In this framework, the up-regulation of a number of transcripts belonging to the LRR-RLK family of proteins is part of this cv. Leccino response. These signaling proteins, whose orthologs are similarly up-regulated in *Xf*-infected grapevine [5] and CVC-resistant mandarin [6], are the first barrier that the plant opposes to pathogen colonization. DNAJ6 chaperone belongs to a class of proteins interacting with HSP70 involved in plant defense response [45]. A similar transcript was up-regulated in the resistant Ponkan mandarin by *Xf* infection [6]. Furthermore the up-regulation of a NAC transcription factor was reported to have a role in modulating plant immune response [46]. This specific defense reaction does not occur in the cv. Ogliarola salentina

Transcriptional changes of cv. Ogliarola salentina consist in the up-regulation of several genes [G-type lectin S-receptor-like serine/threonine-protein kinase SD3-1, ELIP, expansins, LEA and short-chain dehydrogenase/reductase (ABA2)] reported in plants subjected to different stresses with the aim to maintain cell wall structural integrity and protect cell components. Particularly, G-type lectin S-receptor-like serine/threonine-protein kinase SD3-1, expansin 10 and ELIP genes were induced in grapevine, *A. thaliana*, wheat and *Haberlea rhodopensis* in response to water stress or bacterial infections [5, 47–50].

## Conclusions

Our data indicate that *Xfp* is perceived by olive trees of both cultivars, in which it perturbs gene expression. The weaker OQDS symptoms shown in the field by cv. Leccino with respect to cv. Ogliarola salentina might depend on a differential transcriptional response of the two cultivars. A lower bacterial titer of cv. Leccino, suggests that it may also exert a beneficial impact on epidemiology as it may result in a lower rate of *Xfp* transmission by *Philaenus spumarius*, thus helping with the containment of the disease spread. However, these preliminary data need extensive laboratory and field evaluation before a practical exploitation of the cv. Leccino for the management of *Xfp* in olive.

## Methods

### Plants used for the analysis

Olive trees used for the transcriptome analysis were selected on the basis of the following criteria: (i) common age of the trees of the two cultivars; (ii) trees grown under the same management regime and (iii) located in the main outbreak area with consolidated *Xfp* infections; (iv) trees preferably young with reduced risk of concomitant infections caused by other xylem-inhabiting pathogens (i.e. fungal agents associated with sapwood decay of century-old trees [21, 22]); (v) *Xfp*-infected trees showing a clear-cut difference in the disease expression.

Upon numerous field surveys in several districts of the infected area (Salentinian peninsula, Apulia, southern Italy), an olive grove satisfying the aforementioned criteria was selected in the municipality of Gallipoli. This grove consisted of more than sixty 25 years-old olive trees of the cvs Leccino and Ogliarola salentina. For both cultivars the trees consisted in grafted plants grown in contiguous rows. Previous sampling and laboratory tests proved that the infection rate of this grove was close to 100 % (Boscia, unpublished information). Visual inspections revealed that all trees of the cv Ogliarola salentina had severe symptoms of dye-back encompassing the whole canopy, whereas those of cv. Leccino showed leaf scorching and desiccation limited to few twigs. Healthy trees for both cultivars were selected from groves located outside the infected area to avoid the risk of undetectable infections that could interfere with the plant response. The age and phenological state of these controls were comparable with those of the infected trees. From each *Xfp*-infected tree, mature shoots were collected from the sections of the canopy where branches affected by desiccation and dieback were present. Samples consisted in 1- or 2-year-old twigs (*ca.* 0.5 cm in diameter) from which cuttings of 15–20 cm were prepared from the portions close but not yet affected by the withering and desiccation phenomena. Xylem tissue (*ca.* 0.5–1 g) was then recovered, after removing the bark, and processed for DNA or RNA extractions as described below.

### DNA extraction and bacterial quantification

Extraction of total DNAs was performed according to the CTAB protocol reported by Loconsole et al*.* [51]. Absolute quantification of *Xfp* in plant tissues was performed by qPCR according to Harper et al. [52] with TaqMan Fast Advanced Master Mix (Thermo Fisher Scientific, USA) on a CXF 96TM Real time System (BioRad Laboratories, USA). Each sample was run in duplicate and the data were averaged. To obtain a standard calibration curve, serial dilutions of an inactivated bacterial suspension with an initial OD_600_ of 0.5, corresponding to *ca*. 10^8^ CFU/ml, were spiked in total DNAs extracted from xylem tissues of a healthy olive to obtain serial 10-fold dilutions ranging from 10^7^ to 10 CFU/ml. Bacterial quantifications from cvs Leccino (14 plants) and Ogliarola salentina (10 plants) samples were inferred by the standard calibration curve using *Cqs* from qPCR. Reactions were performed on 50 ng/μl total DNAs.

### RNASeq library preparation and sequencing

Transcriptomes of *Xfp*-infected cv Leccino and Ogliarola salentina and corresponding healthy controls, were obtained by sequencing their messenger RNA (mRNA) fractions. Total RNAs were extracted from 1 g of xylem tissues powdered in liquid nitrogen.. Extraction was made in guanidine isothiocyanate buffer according to Foissac et al. [53]. The slurry was extracted once with phenol/chloroform and total RNAs were precipitated with EtOH and resuspended in RPE buffer (RNeasy Plant Mini Kit, Qiagen, The Netherlands). Further purification was made according to manufacturer’s instructions. RNA concentration and quality were evaluated by measuring the absorbance at 260 nm and the absorbance ratio 260/280 with a Nanodrop 2000 spectrophotometer (Thermo Fisher Scientific, USA).

Libraries of xylem-derived mRNAs were synthesized from three infected cvs Leccino (LC1_X, LC2_X, LC3_X) and Ogliarola salentina (OG1_X, OG2_X, and OG3_X) plants and two healthy plants of the same cultivars (LC5_H, LC6_H, OG5_H and OG6_H) (Table 1). A total amount of 3 μg mRNA *per sample* was used for preparation of libraries that were generated using the TruSeq RNA Sample Preparation Kit v2 (Illumina, USA) following manufacturer’s recommendations.

Quality of the synthesized libraries was assessed by the Agilent Bioanalyzer 2100 system (Agilent Technologies, USA). Cluster generation and 50 bp single-end sequencing were performed on a cBot apparatus and HiScanSQ^TM^ platform (Illumina, USA) provided by the SELGE network .

### *De novo* transcriptome assembling

Raw sequence reads were processed using FastX toolkit scripts to remove reads containing only adaptors. *De novo* assembly on these high quality filtered data was done using the SOAPdenovoTrans software [54]. Multiple assemblies were generated per sample library, using a 17–45 range of odd *k-mer* values, in order to capture low-expressed transcripts [55]. Transcripts combined from the multiple *k-mer* assemblies of each library were filtered to select those encoding open reading frames (ORF prediction) and run through the CD-HIT-EST program to remove redundant transcripts sharing 100 % identity [56]. Cultivar-specific transcriptomes were assembled for cvs Leccino and Ogliarola salentina genotypes and an "olive xylem transcriptome” was generated by combining data from both cultivars.

### Differential expression analysis

Raw reads from each library were mapped against assembled transcriptomes using a Bowtie 1.1.2 aligner [57] for differential expression analysis. Raw counts of the mapped reads were extracted from the Bowtie alignment of each library to estimate the transcript abundance using the SeqMonk software .

The DESeq2 Rpackage 1.10.1 from Bioconductor [58] was used to statistically evaluate the relationships among different libraries and the expression level of the transcripts. DESeq2 performs statistical analysis (calculation of sample-to-sample distances; PCA) on raw counts to execute normalization, variance estimation and differential expression. The software uses a model based on negative binomial distribution by adjusting the obtained P-values by the Benjamini and Hochberg’s procedure for controlling the false discovery rate. Stringent parameters consisting on a >2fold expression ratio and a false discovery rate (FDR) of p-values less than 0.001, were used in DESeq2 to identify differential expressed genes.

Differential gene expression was analyzed between each *Xfp*-infected and corresponding healthy olive cvs (LC_X *vs* LC_H and OG_X *vs* OG_H), between *Xfp*-infected plants of the two cvs (LC_X *vs* OG_X) and between healthy plants of the two cvs (LC_H *vs* OG_H). Parallel analysis with SeqMonk allowed extracting genes that exceed the basic threshold of system destabilization, providing an additional list of DEGs. Comparison of DESeq2 and SeqMonk selected DEGs allowed selecting common genes whose change could be more strongly related to the infection by *Xfp*.

Differentially expressed transcripts found common between DESEq2 and SeqMonk were determined and used for further analysis.

Annotation of differentially expressed transcripts was conducted by sequence similarity searches against the NCBI non-redundant protein sequence database (nr). BLASTX E-value cut-off was set at 10^−5^.

Moreover, a FASTA file with all DEGs was generated and transcripts categorized by Mercator for Bincode mapping, and MapMan v.3.5.1 [59] for pathway analysis. Default parameters were used and JGI Chlamy Augustus models, TIGR5 rice proteins, InterProScan, and a default BLAST cut-off of 80 were chosen.

The overlap between different sets of differentially expressed transcripts was generated by the Venn diagram generator using VENNY, an interactive tool for comparing lists with Venn diagrams [60].

### Functional annotation and GO enrichment analysis

A gene set containing the 22,373 unique transcripts longer than 700 nt of the "olive xylem transcriptome” were locally aligned against the *Viridiplantae* subset of the NCBI nr database (downloaded on 03-21-2016) using BLASTX with a significance cut-off E-value <10^−6^. The Blast2GO suite (http://www.blast2go.com) [61] was used for functional annotation of the transcripts and enrichment analysis based on the gene ontology (GO) terms. All 512 up- and downregulated DEGs identified in the comparison among cv Leccino *vs* cv Ogliarola Xfp-infected plants (Additional file 6) were tested for GO enrichment using the Fisher's exact test, in the Blast2GO software.

Enrichment analysis for over- or underrepresentation of GO terms were done for all DEGs compared to all annotated genes in the high confidence gene set, using a cut-off threshold of FDR ≤ 0.05. The analyses were performed for the three ontology levels, biological processes (P), molecular function (F) and cellular component (C).

### Quantitative RT-PCR

Eleven genes, identified by RNA-Seq to be differentially expressed in cvs Leccino and Ogliarola salentina in response to *Xfp* infection, were selected for validating DE analysis by RT-qPCR. Primers for LRR-LRK, PME, ELIP, FAR-RED, EXP, FLA, LAC17, LAC12, ABA2, CRK8/10, CRK6 (Additional file 8) were designed using Primer-Blast tool, available at NCBI website, and checked *in silico* for their specificity to target transcripts. Total RNAs were isolated with RNeasy Plant Mini Kit (Qiagen, USA), digested with RQ1 RNase-Free DNase (Promega Corporation, USA) and reverse transcribed with M-MLV reverse transcriptase (Thermo Fisher Scientific, USA). RT-qPCR was carried out on a CFX96™ Real-Time System (Bio-Rad Laboratories, USA) using SYBR Select Master Mix for CFX (Thermo Fisher Scientific, USA), according to manufacturer’s protocol. The following thermal cycling profile was used for all RT-qPCR reactions carried out in this study: 50 °C for 2 min, 95 °C for 2 min, 40 cycles of 95 °C for 15 s, 58 °C for 15 s and 72 °C for 20 s. Primer specificity of all evaluated genes was confirmed by checking the absence of nonspecific amplifications as resulting from melting curve analysis assessed from 65 °C to 95 °C. The three infected and two healthy plants of cvs Leccino and Ogliarola salentina were tested and each sample was amplified in three technical replicates. The efficiency of each set of primers was estimated using the Real-time PCR Miner v. 4.0 software [62], which allows calculating the kinetics of individual PCR reactions.

In order to find a reference gene to normalize the RT-qPCR results, five olive endogenous housekeeping genes, namely β-ACT, UBQ, UBCE, EF1 α and EF2, were preliminary tested. Primers for β-ACT were those by Schilirò et al. [63], whereas the other sets were designed in this study (Additional file 8). All amplifications were conducted as described above.

Data resulting from RT-qPCR were processed with CFX Manager Software (Bio-Rad Laboratories, USA) and used to estimate relative expression (RE) for each gene target against the reference genes, in cvs Leccino and Ogliarola salentina separately. RE was calculated according to the formula:

*RE* = (1 + *E*) − ^*ΔΔCt*^ where RE = Relative Expression; E = Efficiency; Ct = Cycle Threshold. E and Ct are expressed as average values between three technical replicates. Based on Livak and Schmittgen [64] criteria, modified to account for differences in efficiency between target and reference genes, although comprised in the optimal range of 5 %. REs for each biological replicate were averaged and expressed as fold-change ratio (infected:healthy), in cvs Leccino and Ogliarola salentina separately. To verify statistical correlation between fold-change ratio, as obtained by DESeq2 and RT-qPCR quantifications, a linear regression analysis was computed, and R^2^ coefficient of determination was calculated and graphically plotted.

Statistical analysis of expression levels of each transcripts was performed with the SPSS 23.0 software program (IBM, Armonk, NY, USA). Comparisons among numeric data sets was conducted using the one-way ANOVA followed by Tukey’s HSD test. Statistical significance was accepted for p-values < 0.05 α-level.

## Abbreviations

ABA, abscisic acid; ABA*,* abscissic acid; ABA2, short-chain dehydrogenase/reductase; BLAST, Basic Local Alignment Search Tool; CEGs*,* core eukaryotic genes; CFU*,* Colony Forming Units; CoDiRO*,* "Complesso del Disseccamento Rapido dell’Olivo"; CRK, cysteine-rich RLKs; CWDEs*,* cell wall degrading enzymes; DAMP*,* damage-associated molecular patterns; DEGs*,* differentially expressed genes; EF1a, elongation factor 1-alpha; EF2, elongation factor 2-like; EFR*,* elongation factor Tu receptor; Egase*,* endo-1,4-b-glucanase; ELIP, early light-induced proteins; ETI*,* effector triggered immunity; EXLA, expansin-like A; EXLB, expansin-like B; EXPA, expansin A; EXPB, expansin B; FAR-RED, far-red impaired response 1; FDR*,* false discovery rate; FLA*,* Fasciclin-like arabinogalactan; FLS2*,* flagellin-sensitive 2; HG*,* homogalacturonan; LAC17, laccase 17; LC_H*,* Leccino healthy; LC_X*,* Leccino infected; LEA, late embryogenesis abundant protein; MAMP*,* microbe-associated molecular patterns; mRNAs*,* messengers RNA; NCBI, National Center for Biotechnology Information; OG_H*,* Ogliarola salentina healthy; OG_X*,* Ogliarola salentina infected; OQDS*,* olive quick decline syndrome; PCA*,* principal component analysis; PG*,* polygalacturonase; PL, pectin lyases; PME*,* pectin methylesterase; PMEIs*,* PME inhibitor; PRRs*,* pattern recognition receptors; PTI*,* pathogen triggered immunity; qPCR*,* quantitative Polymerase Chain Reaction; RLK*,* receptor-like kinases; RLP*,* receptor-like proteins; RNA*,* Ribonucleic acid; RNASeq*,* RNA sequencing technology; rRNA*,* ribosomal RNA; TSA, Transcript Shotgun Assembly; TuMV, *Turnip mosaic virus*; UBCE, ubiquitin-conjugating enzyme E2; UBQ, polyubiquitin; *Xf,* Xylella fastidiosa; *Xfp, Xylella fastidiosa* subsp*. pauca*; *Xoo, Xanthomonas oyzae* pv *oryzae*; XyG*,* xyloglucan; β-ACT, beta actin

## Additional files

Additional file 1: Length distribution of transcripts of the combined “olive xylem transcriptome”. (PPTX 255 kb) Additional file 2: Mapping of transcripts from the “olive xylem transcriptome” to a set of Core Eukaryotic Genes (CEGs). Percentages of mapped transcripts toward the four CEGs groups are reported. (PPTX 46 kb) Additional file 3: Differentially expressed transcripts in cv. Leccino infected by *Xylella fastidiosa*. Each unique transcript is categorized by Mercator and Log2 fold expression values calculated by DESeq2 analysis. Transcript categorization was further verified by BLASTX search against the non-redundant protein database. Up- and down-regulated transcripts are in separate excel sheets. (XLSX 138 kb) Additional file 4: Differentially expressed transcripts in cv Ogliarola salentina infected by *Xylella fastidiosa*. Each unique transcript is categorized by MapMan and Log2 fold expression values calculated by DESeq2 analysis. Transcript categorization was further verified by BLASTX search against nr database. Up- and down-regulated transcripts are in separate excel sheets. (XLSX 10155 kb) Additional file 5: Differentially expressed transcripts between healthy cv. Leccino and Ogliarola salentina plants. (XLSX 29 kb) Additional file 6: Differentially expressed transcripts in comparison of cv Leccino vs Ogliarola salentina infected by *Xylella fastidiosa*. Each unique transcript is categorized by MapMan and Log2Fold expression values calculated by DESeq2 analysis. Transcript categorization was further verified by BLASTX search against nr database. Up- and down-regulated transcripts of both cultivars are in separate excel sheets. (XLSX 10197 kb) Additional file 7: GO enrichment analysis of the 512 DEGs between Xfp-infected cv. Leccino and Ogliarola salentina plants. (XLSX 294 kb) Additional file 8: Graphic plots showing the range of Log2 fold change values as they appear in the RNASeq quantitation using SeqMonk. *Per probe* normalisation was applied to the graph to obtain a wider dynamic range. This has the effect of emphasising the changes between conditions whilst ignoring the magnitude of the differences. a) Log2 fold change of candidate transcripts of housekeeping genes; b) Log2 fold change of candidate transcripts of target genes. (PPTX 3708 kb) Additional file 9: Oligonucleotides of selected target and reference genes. Transcripts used for design and corresponding lengths are reported. Categorization of transcripts by BLASTX against the NCBI non-redundant protein sequences database, BLASTN against the Transcriptome Shotgun Assembly databases and orthologous *A. thaliana* genes are indicated. (XLSX 21 kb) Additional file 10: ANOVA one-way comparing the expression levels of selected transcripts obtained by RT-qPCR among the four groups (LC_H, LC_X, OG_H, OG_X). Post hoc multiple comparisons using the Tukey HSD test of individual averages of expression levels. A p-value lower than 0.05 (in bold) indicates that expression levels of single transcripts are significantly different. Significance level was set at 5 % (α = 0.05) for F- and p-values. Df indicates the degree of freedom between groups. (XLS 48 kb)
