# Supplementary figures and images for: Transcriptome profiling of two olive cultivars in response to infection by the CoDiRO strain of Xylella fastidiosa subsp. pauca
Source: BMC Genomics. 2016 Jun 27;17:475. doi: 10.1186/s12864-016-2833-9 (PMC4924284; doi:10.1186/s12864-016-2833-9)

## Slide 1
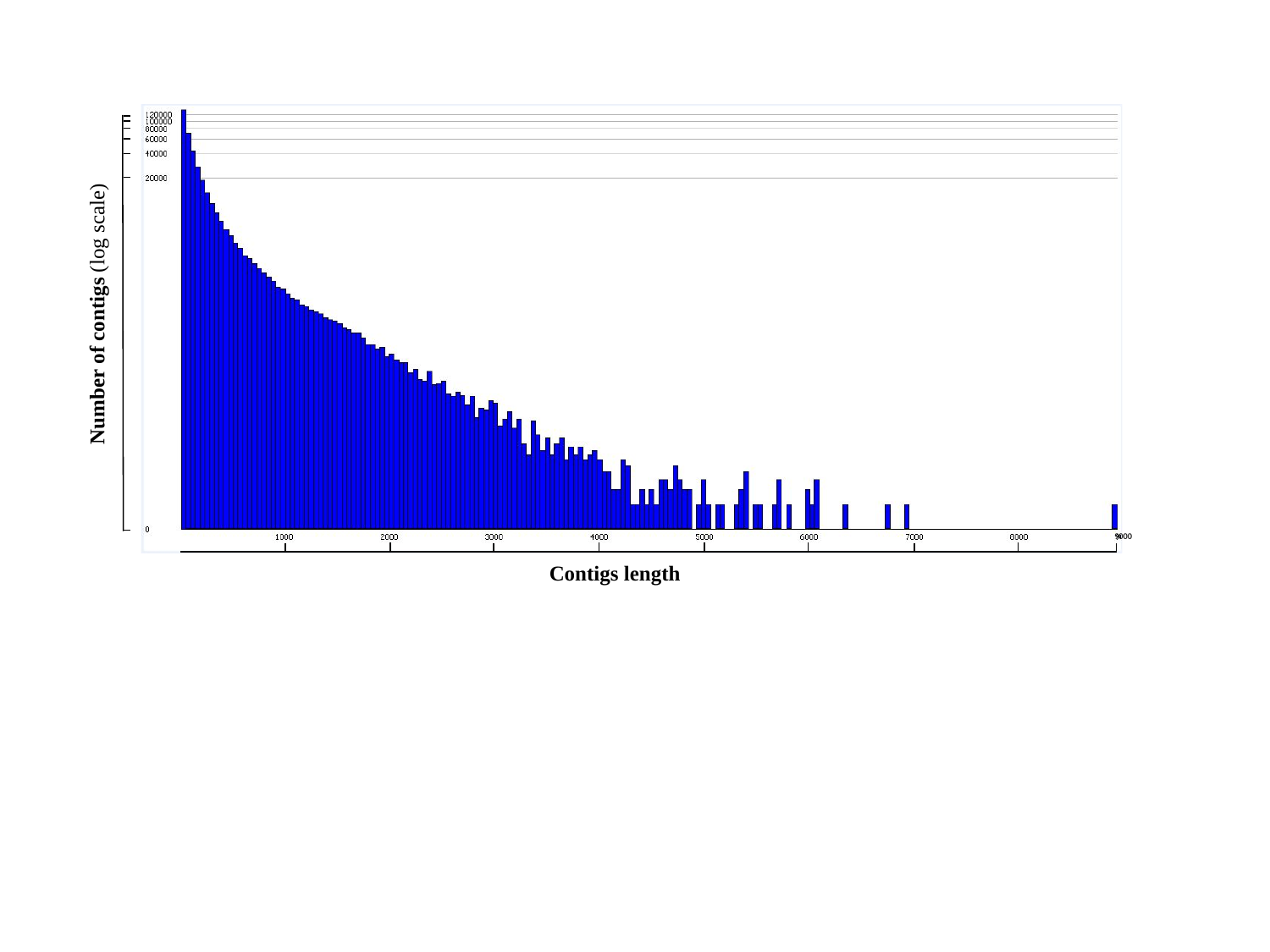

9000
Number of contigs (log scale)
Contigs length

Supplement: Additional file 1: — Length distribution of transcripts of the combined “olive xylem transcriptome”. (PPTX 255 kb) [file 12864_2016_2833_MOESM1_ESM.pptx]

## Slide 1
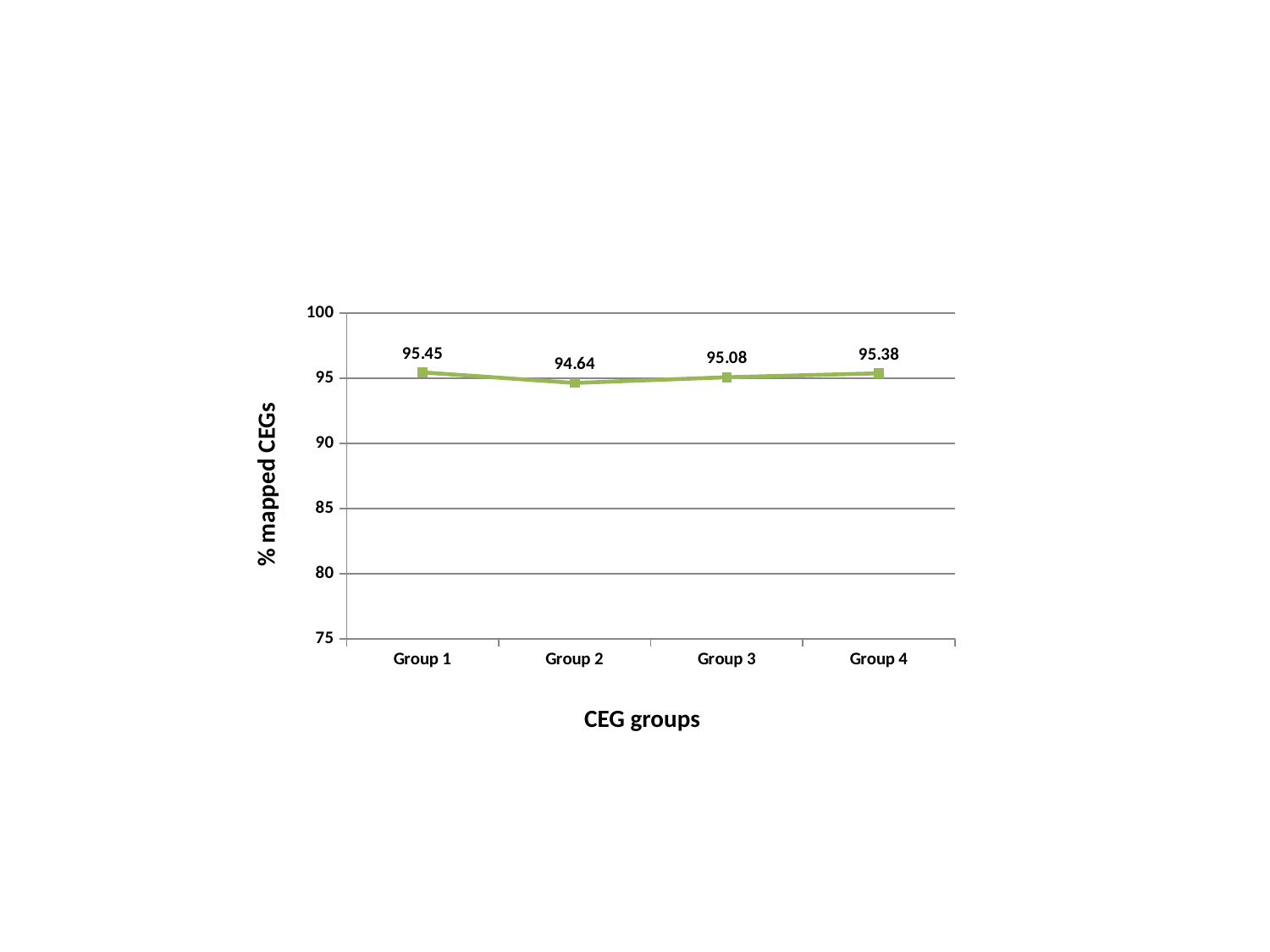

### Chart
| Category | %Completeness |
|---|---|
| Group 1 | 95.45 |
| Group 2 | 94.64 |
| Group 3 | 95.08 |
| Group 4 | 95.38 |% mapped CEGs
CEG groups

Supplement: Additional file 2: — Mapping of transcripts from the “olive xylem transcriptome” to a set of Core Eukaryotic Genes (CEGs). Percentages of mapped transcripts toward the four CEGs groups are reported. (PPTX 46 kb) [file 12864_2016_2833_MOESM2_ESM.pptx]

## Slide 1
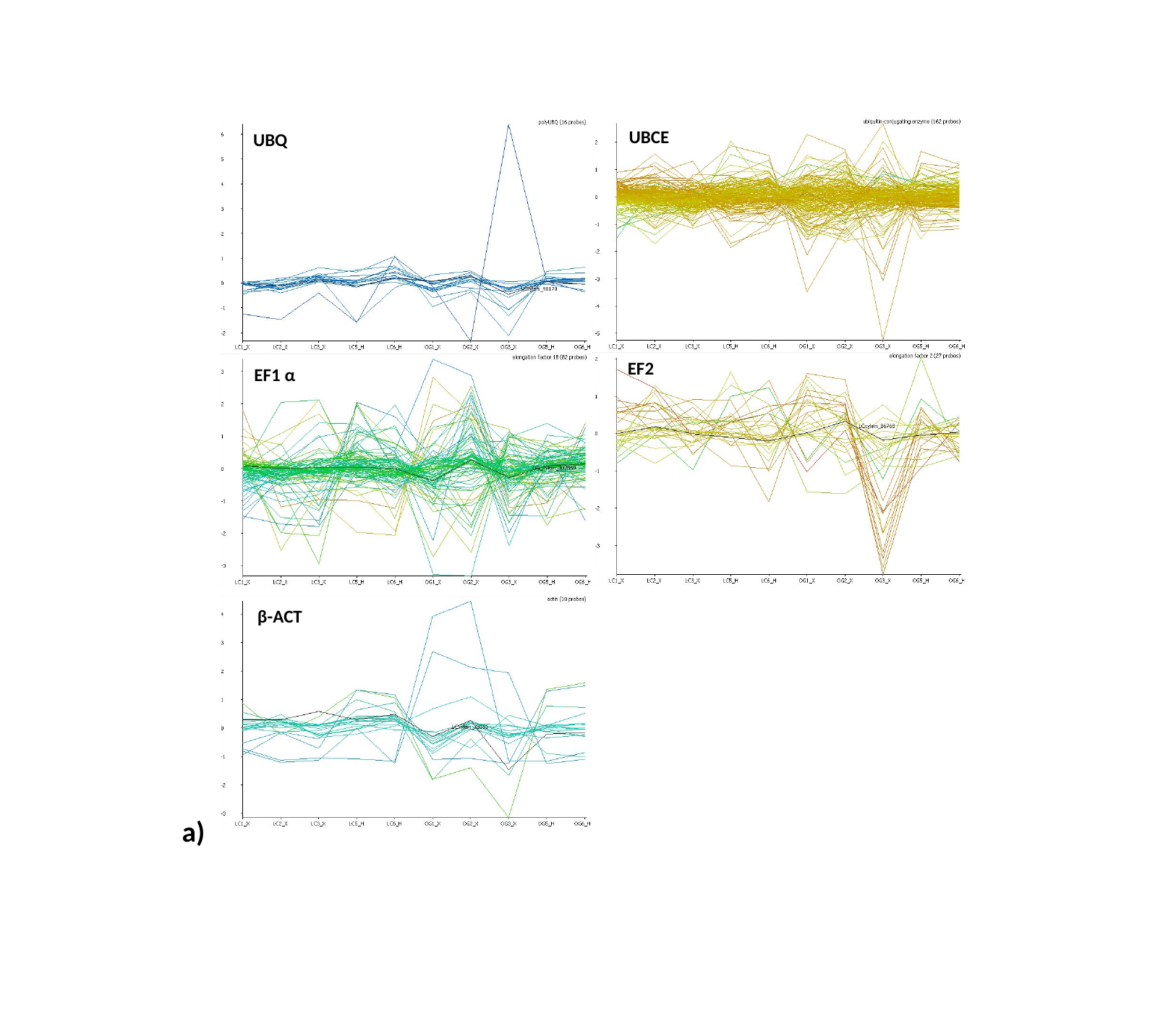

UBCE
UBQ
EF2
EF1 α
β-ACT
a)

## Slide 2
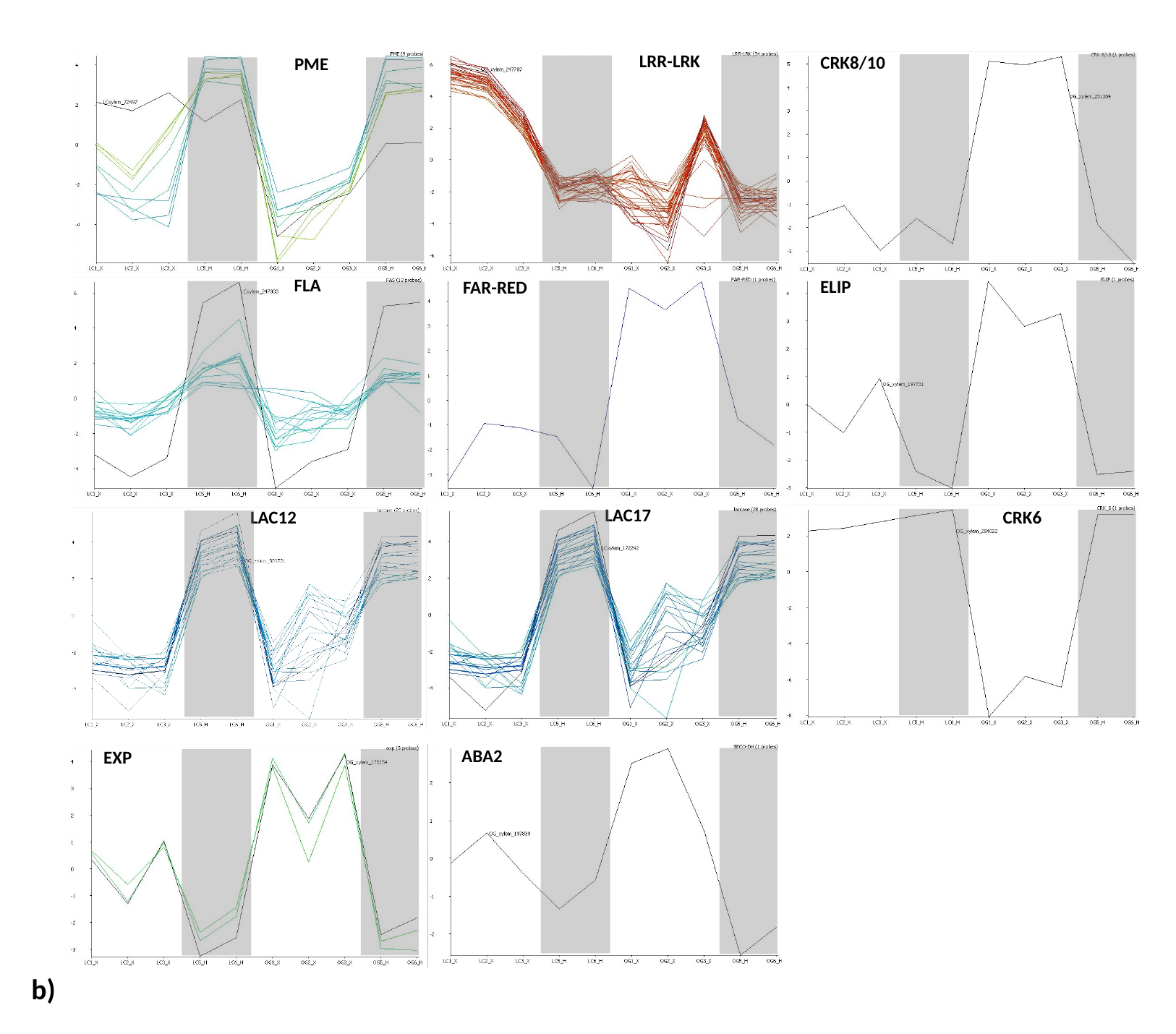

LRR-LRK
CRK8/10
PME
FLA
ELIP
FAR-RED
LAC17
CRK6
LAC12
ABA2
EXP
b)

Supplement: Additional file 8: — Graphic plots showing the range of Log2 fold change values as they appear in the RNASeq quantitation using SeqMonk. Per probe normalisation was applied to the graph to obtain a wider dynamic range. This has the effect of emphasising the changes between conditions whilst ignoring the magnitude of the differences. a) Log2 fold change of candidate transcripts of housekeeping genes; b) Log2 fold change of candidate transcripts of target genes. (PPTX 3708 kb) [file 12864_2016_2833_MOESM8_ESM.pptx]
